# Violet Anthraquinone for Expanding the Color Palette of Electrochromes with Three Discrete Colors and Full Color Bleaching

**DOI:** 10.3390/molecules31050879

**Published:** 2026-03-06

**Authors:** Ilies Seddiki, Thierry Maris, W. G. Skene

**Affiliations:** 1 Chemistry Department, Université de Montréal, 1375 Avenue Thérèse-Lavoie-Roux, Montreal, QC H2V 0B3, Canada; 2 X-Ray Diffraction Laboratory, Chemistry Department, Université de Montréal, 1375 Avenue Thérèse-Lavoie-Roux, Montreal, QC H2V 0B3, Canada; 3 Institut Courtois, Université de Montréal, Montreal, QC H3C 3J7, Canada

**Keywords:** anthraquinone, chromophore, electrochromism, spectro-electrochemistry, colorimetry, structure–property relationship

## Abstract

An anthraquinone chromophore displaying a vivid violet color in solution was synthesized and it was thoroughly characterized both spectroscopically and electrochemically, along with its X-ray crystallography. Single crystal X-ray analysis of the chromophore revealed a nearly planar π-conjugated framework with short intermolecular contacts. Cyclic voltammetry revealed two consecutive one-electron reductions, corresponding to the formation of its radical anion and dianion. The spectroelectrochemistry of the chromophore confirmed two distinct and reversible color changes with the stepwise electrochemical reduction. These were quantified via the CIE L a* b* color space. Large optical differences (98%) between the bleached and colored states were observed along with a coloration efficiency of 698 cm^2^/C. These parameters confirm the anthraquinone is an ideal electrochrome: capable of reversibly switching its colors with applied potential. The three color changes and color bleaching associated with the neutral, radical anion, dianion, and cation, respectively, are also of interest for extending the palette of colors of molecular electrochromes toward panchromatic color tuning with molecular structure for use in smart windows and displays.

## 1. Introduction

Anthraquinones are widely recognized for their rich reversible electrochemistry that enables a range of applications. Indeed, they play important roles as polymerization inhibitors [1], dyes [2], and pigments [3]. Anthraquinones have also gone through a resurgence in activity as electroactive materials for energy storage owing to their capacitive two-electron storage [4]. The use of anthraquinones in energy storage is further spurred on by their sustainability, being derived from naturally recurring resources such as ferns/conifers [5] and marine fungi [6]. Integrating such sustainable materials into energy storage contributes to advancing the environmental circularity of electricity generation. The additional virtue of anthraquinones is their degree of conjugation that gives rise to visible absorptions and emissions [7,8,9,10]. Both the color and emission wavelength can be tailored contingent on molecular structure [11]. Leveraging both the visible color and electrochemistry make anthraquinones ideal candidates for electrochromics: change in color with applied bias. Smart windows for automotive and architectural uses [12], displays [13,14], and anti-counterfeiting [15] are tangible applications that have benefited from the reversible color change with applied potential of electrochromes [16].

**Figure 1:**
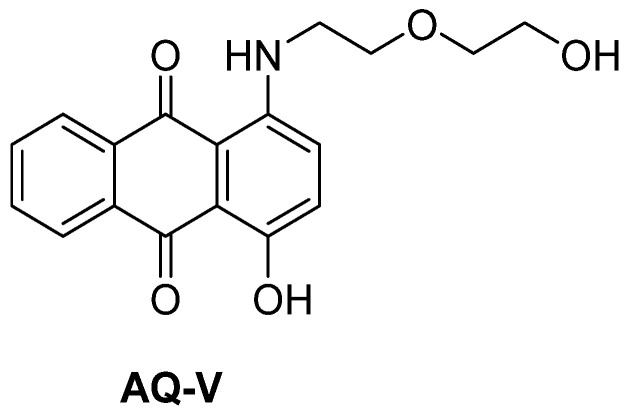
Structure of anthraquinone molecular electrochrome prepared and evaluated.

Recent developments in π-conjugated anthraquinones have highlighted their potential for multicolor electrochromic devices, sensors, and energy-storage systems [17,18,19]. Although anthraquinones are poised to be ideal electrochromes for such applications owing to their electrochemical reduction and reversible visible colors, they are often overlooked. They are advantageous over their molecular anodic counterparts because of the greater chemical stability of the electrochemical-mediated intermediates, resulting in no undesired electrochemically induced byproducts [20,21]. This has the benefit of maintaining the color of the different states with repeated cycles of applied potential [22,23]. The two reduced states of anthraquinones can further be exploited to give rise to three discrete colored states: neutral, radical anion, and dianion concomitant with color-bleaching of the oxidized state. This contrasts with the anodic counterparts that typically have only two colored states [24,25]. Despite this, using anthraquinones as electrochromes is relatively underused. Bearing this in mind, it is of interest to understand the effect of molecular structure on both the anthraquinone’s electrochemistry and the possible color changes with applied potential. This knowledge could be leveraged to ultimately rationally design molecular electrochromes having targeted colors for both the bleached and colored states. Toward providing such insight, we prepared the anthraquinone (**AQ-V**; Figure 1). The design elements of **AQ-V** include the amine that serves three roles: (i) an electron donor, (ii) hydrogen donor for hydrogen bonding and (iii) *N*-alkylation. The collective electronics and hydrogen bonding were expected to shift the otherwise off-yellow color of the native anthraquinone into the visible region [26]. This would be advantageous for further ensuring the absorption of the corresponding radical anion and dianion would also absorb in the visible, each with discrete colors. Collectively, these attributes would potentially confer three colored states to the electrochrome corresponding to its neutral, radical anion and dianion states, and color-bleached cation, whereas *N*-alkylation would ensure solubility of **AQ-V** in a range of common solvents. The electrochromism of **AQ-V** is herein presented along with its X-ray crystallography to evaluate the effect of the electronic group substitution on the color without affecting the desired electrochemical redox activity. To reinforce the suitability of **AQ-V** as a viable electrochrome, its optical contrast, coloration efficiency, and switching kinetics are also herein presented.

## 2. Results

### 2.1. Crystal Structure

**Figure 2:**
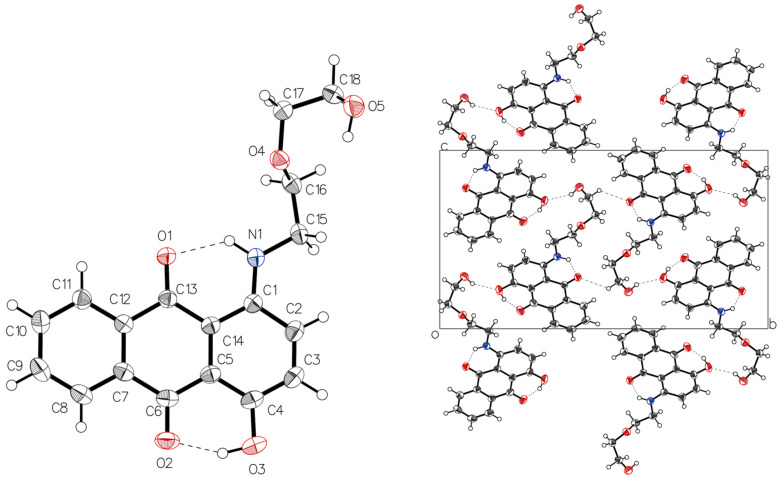
ORTEP diagram of the single X-ray crystal structure of **AQ-V** with the ellipses drawn at 50% probability and the hydrogens arbitrarily drawn (**left**). Projection along the *a*-axis of the unit cell showing extended packing of **AQ-V** illustrating both the intra- and intermolecular hydrogen bonds as dashes (**right**).

**Figure 3:**
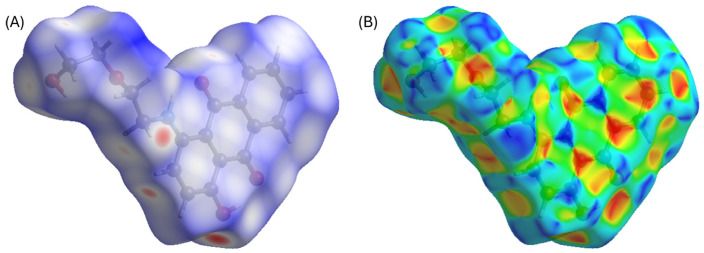
(**A**) **AQ-V** representation of d_norm_ with red dots indicating sites of intermolecular interactions. (**B**) **AQ-V** representation of shape index with adjacent blue and red triangles indicating π-π stacking location. Both images calculated with Crystal Explorer [27,28].

**AQ-V** crystallized in the *P*2_1_/*n* monoclinic space group. The crystal structure revealed a complex network of hydrogen bonds. The carbonyl acted as a hydrogen-bond acceptor, promoting intramolecular interactions with the neighboring amine and the alcohol (left panel Figure 2). Indeed, two intramolecular hydrogen bonds, along with a total of three intermolecular hydrogen bonds involving O1, O2, O3 occurred between different molecules (Table S1). These intramolecular contacts enhanced the hydrogen-donor character of the secondary carbon adjacent to the amine. This, in turn, formed intermolecular interactions with the oxygen of the carbonyl on neighboring molecules. The overall crystal packing was further stabilized by intermolecular hydrogen bonds involving the secondary carbon and the hydroxyl of the rigid aromatic core. Additional stabilization arises from interactions between the carbonyl and hydroxyl groups along the alkyl side chains. The intermolecular contacts can readily be visualized as red dots in the d_norm_ representation of Figure 3A. These combined interactions led to a slight deviation from coplanarity of the tricyclic aromatic, reducing the π–π stacking overlap between adjacent molecules.

π–π stacking occurred in the solid-state packing. This is visually evident in the shape index calculated in Crystal Explorer [27,28]), according to the well-defined adjacent triangles forming an hourglass shape via the tip-to-tip (Figure 3B). The complementary colors, red and black, correspond to concave and convex regions, respectively, on the electrostatic potential. These interactions occur between two of the three anthraquinone aromatics of adjacent molecules. These interactions are quantifiable and are summarized in the crystallographic table (Table S2). In short, several near-parallel slipped π–π contacts occur between ring centroids with the shortest centroid-to-centroid distances of 3.8379(9) and 3.8755(9) Å [29]. The collective crystallographic data confirm that multiple contacts, including hydrogen bonding and π–π stacking, occur despite the simple structure and the alkyl chain that was expected to disrupt these interactions.

### 2.2. Electrochemistry

**Figure 4:**
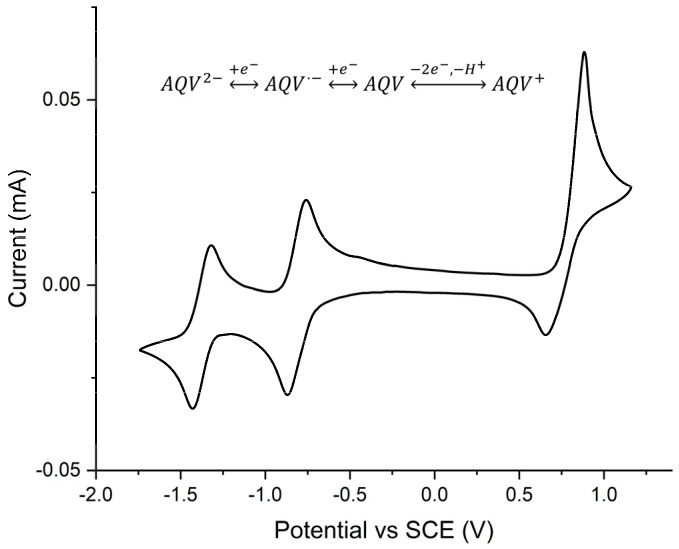
Cyclic voltammogram of **AQ-V** measured in anhydrous DMF at 50 mV/s with Bu_4_NClO_4_ electrolyte. Inset: corresponding electrochemically generated intermediates of **AQ-V**.

The overarching property that is required of anthraquinones for their use as electrochromes is electrochemical activity. Their electrochemically induced intermediates must also be persistent and do not react in ways that would otherwise result in both the depletion of the transients and the neutral state. This would result in inconsistent color changes and mixed absorptions rather than desired discrete colors. Anhydrous DMF was the choice solvent for cyclic voltammetry to evaluate the chemical inertness of the intermediates because of its large window of stability for evaluating both the expected anodic and cathodic processes of **AQ-V**. Moreover, its electrochemical inertness is biased to the negative potentials compared to other solvents [30,31]. This is important for assessing the two expected electrochemical reductions of **AQ-V**. As expected, owing to the anthraquinone core, **AQ-V** underwent two discrete reductions at E_1/2_ = −0.81 and −1.37 V vs. SCE (Figure 4). These were assigned to the consecutive one-electron reductions to form the radical anion and the dianion according to their similar peak intensity and identical peak current to the internal ferrocene standard. The identical peak current of the forward and reverse waves of the cathodic processes illustrates the chemical stability of the radical anion and dication within the timeframe of the measurement. Consistent cyclic voltammograms were measured during 300 cycles of reversibly applied potential between +1.1 and −1.8 V. Indeed, no change in the voltammograms was observed after the extended cycling compared to the original voltammogram (Figure S3). This contrasts with the anodic process forming the radical cation whose reverse wave was 1/3 the intensity of the corresponding forward wave (E_1/2_ = 0.77 V). Based on the relative peak current, the anodic process involves two simultaneous one-electron oxidations occurring at the amine. The amino radical cation formed from a one-electron oxidation rapidly deprotonates followed by simultaneous subsequent one-electron oxidation. Deprotonation of the amino radical cation is chemically irreversible as per the cyclic voltammogram. This aside, the advantage of the amine conjugated with anthraquinone is it opens the anodic channel that is otherwise not active with native anthraquinones.

The cyclic voltammetry data confirmed both the cathodic and anodic processes of **AQ-V**. It also provided information about the chemical stability of the radical anion and dianion intermediates produced, an important electrochromic requirement. The peak-to-peak separation (ΔE_p_ ≈ 110 mV) concomitant with the linear correlation of the peak current with the square root of the scan speed confirmed both the electrochemical processes are diffusion-controlled and quasi-reversible [32].

### 2.3. Spectroelectrochemistry

The other requisite for an electrochrome is the reversible color change with applied potential. This was evaluated by monitoring the change in absorption with applied potential. The spectroelectrochemistry was done in situ using a commercial low-volume cell and an electrode with a 19-well honeycomb electrode. Although the measurements were done in solution, both the low volume of the wells and the narrow optical path of the cell limit diffusion of the electrochrome. Therefore, **AQ-V** can be completed converted to its corresponding intermediates with applied potential. The large number of wells of the working ceramic honeycomb creates a sufficiently large area to track the change in absorption spectroscopically.

**Figure 5:**
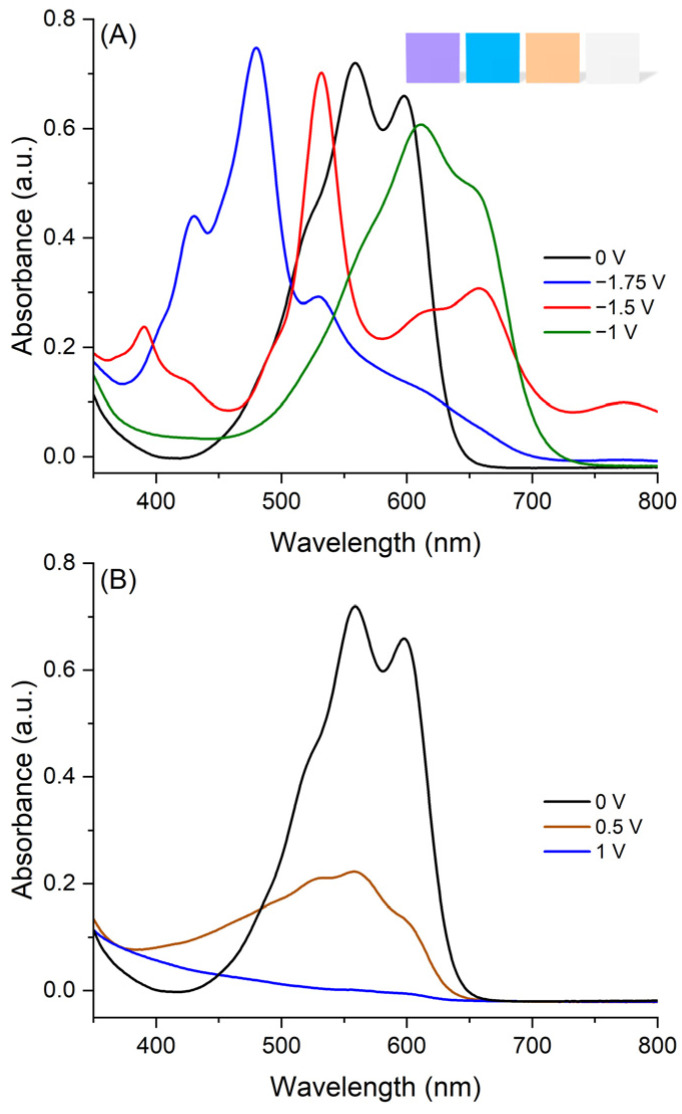
Spectroelectrochemistry of **AQ-V** with negative (**A**) and positive (**B**) potentials in anhydrous DMF with Bu_4_NClO_4_. Inset: representative perceived colors of **AQ-V** in the neutral, radical anion, dianion, and radical cation (from **left** to **right**).

The change in absorption was monitored after applying a given potential for a set period of time and the resulting spectrum measured. The potential applied was maintained during the acquisition of the absorption spectrum to ensure the spectrum recorded was the steady-state product and not the continuous formation of various species. The negative potential was gradually increased to convert the original **AQ-V** to the radical anion and then to its dianion. The resulting change in the absorption spectrum is found in Figure 5A. Of importance is the unique absorption of each state corresponding to a unique color for the original **AQ-V**, its radical anion and dianion. Indeed, the original absorptions of **AQ-V** at 560 nm and its minor absorption at 600 nm were replaced with the absorption at 530 nm, corresponding to the radical anion. This absorption was depleted and it was replaced with the absorption of the dianion at 480 nm. The corresponding perceived colors changed from violet for **AQ-V** to cyan and yellow for the radical anion and the dianion, respectively (inset Figure 5A). The original violet color of **AQ-V** could also be bleached with a positive potential. Indeed, the absorption of the cation in the visible region is completely bleached with its absorption shift to the UV (orange line Figure 5B).

**Figure 6:**
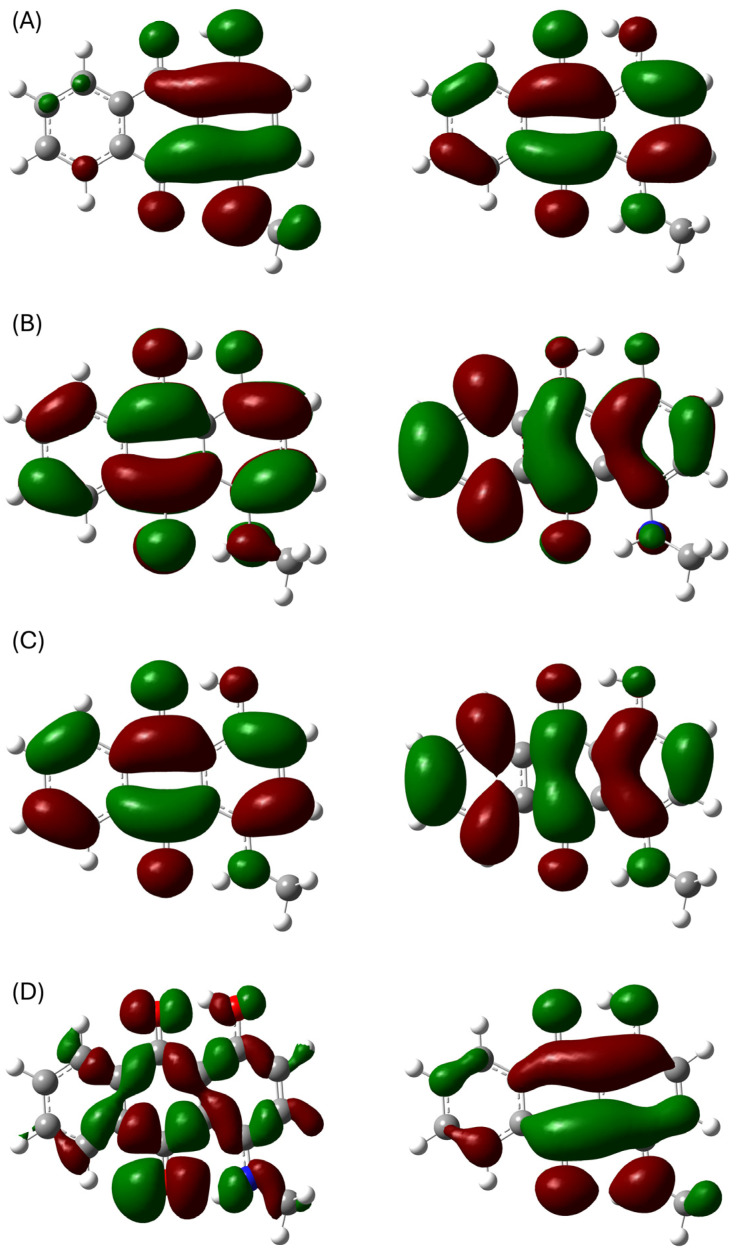
Highest (**left**) and lowest (**right**) occupied Natural Transition Orbitals calculated of the neutral **AQ-V** (**A**), dianion (**B**), α-spin of radical anion (**C**), and β-spin of radical cation (**D**).

To corroborate the electrochemically induced spectral changes, the electronic transitions of the various states were calculated. This included the neutral **AQ-V** and its corresponding radical anion, radical cation, and dianion. The ωB97X-D long-range hybrid correlated functional was chosen because it considers both short- and long-range interactions and it is capable of modeling hydrogen bonding. The latter is ideal given the two intramolecular hydrogen bonds (vide supra). The same trend in spectral changes was calculated (Figure S6) as seen experimentally, according to the principal transitions. The absorptions indeed progressively blue-shifted upon progressing from the neutral **AQ-V** to the radical anion, dication, and the radical anion. The calculations also gave insight into the orbitals in these transitions. The Natural Transition Orbitals were calculated because they consolidate the various molecular orbitals into a single set of orbitals for the given transition [33]. For example, the HOMO of the neutral state of **AQ-V** is concentrated on both the central and substituted aromatics. This contrasted with its corresponding LUMO that was delocalized over the entire anthraquinone. This is responsible for intramolecular transfer of the lowest energy transition (Figure 6A). Both the orbitals of ground and lowest excited states of the corresponding intermediates were similarly distributed across the anthraquinone. It is worth noting that the α-spin orbitals were principally involved in the lowest energy optical transition of the radical anion whereas they were the β-spin orbitals for the radical cation.

**Figure 7:**
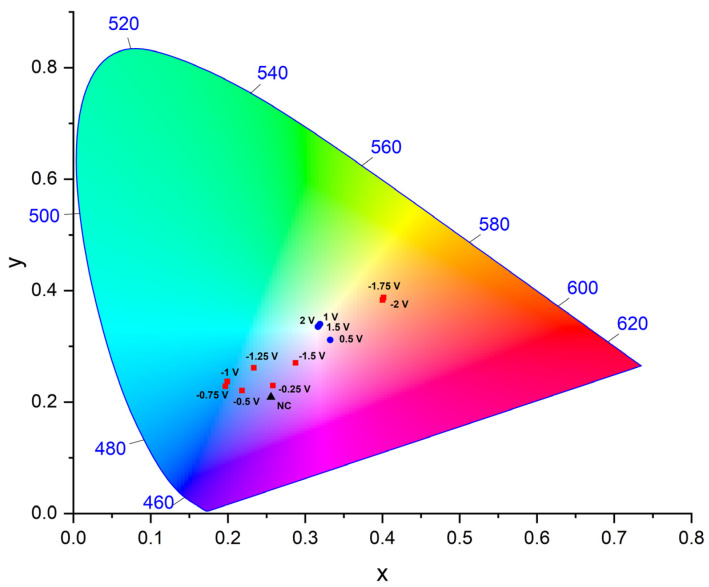
CIE color coordinates of **AQ-V** with applied potential; NC = no current.

Both the perceived color and the absorption changes with applied potential confirm **AQ-V** is suitable for electrochromic use. The color changes with applied can be separated into perceived primary colors according to the CIE coordinates. The convention divides the absorption into its lightness (L), red/green (a*), and blue/yellow (b*) contributions. The values can then be plotted according to a CIE 1976 diagram (ISO 11664-4:2019 [34]) to visually track the composition of each color contribution to the spectrum. Here, the neutral **AQ-V**, without applied potential, is taken as the reference to benchmark the change in color with applied potential. With the applied negative potential generating the radical anion, the lightness remains the same along with the blue contribution to the spectrum. Meanwhile, the red component is replaced with green, according to the negative a* (Table 1). The coordinates of the dianion are equally different than both the original **AQ-V** and the dianion. Of noteworthiness is the lightness that increased while the green contribution was eliminated and it was replaced with red. These CIE changes shift the perceived color of the dianion toward the orange spectrum (Figure 7). The color is nearly bleached with the applied potential, corresponding to near-perfect white in the diagram. Collectively, the applied potential significantly varies the color from blue to yellow, according to the diagram, being an ideal electrochromic response.

### 2.4. Electrochromic Performance

**Figure 8:**
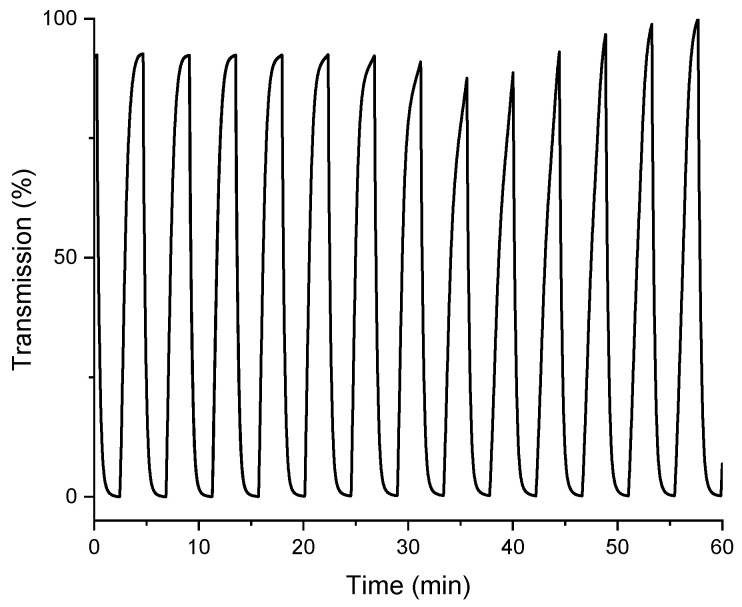
Change in percent transmission of **AQ-V** dianion monitored at 530 nm with applied potentials of −2 V and −1.5 V.

The electrochromic performance of **AQ-V** was further evaluated by monitoring the change in transmission of both the radical anion and the dianion over repeated cycles of reversibly applying the potential. This provides information about the colorfast of both the bleached and colored states. Variations in either the original **AQ-V** and its electrochemically induced colored state by irreversible and unwanted reactions would be evident as a decrease in the optical difference (ΔT%). As seen in Figure 8, the percent transmission of the two states is constant at ca. 98% over 60 min. of continuous switching between the neutral and radical anion and 98% when switching between the radical anion and dianion.

The coloration efficiency (CE; Equation (S1) in Supplementary Materials) is an equally important performance metric of electrochromes. This correlates with the amount of charge per area required to induce the optical change. CE is typically monitored at a unique wavelength. The CE of the radical anion was 506 cm^2^/C at 530 nm while the corresponding dianion was 698 cm^2^/C at λ = 480 nm. To frame this performance value, the best CE of an inorganic electrochrome reached 55 cm^2^/C [35]. Similarly, the most highly performing organic electrochromes that are derived from viologen [36], PEDOT:PSS [37,38] and aniline [39] have a maxima CE of 680 cm^2^/C. According to the calculated CEs, the color of the cation is easier to produce than the dianion, according to the CE.

Chronoamperometric measurements further revealed reversible color switching with coloration and bleaching times (t_colored_, t_bleached_) of 21 s and 82 s for the first reduction and 15 s and 125 s for the second reduction, respectively. The switching kinetics are limited by slow diffusion to the electrode in contrast to fast electron transfer in solid-state devices. This aside, after 60 min of cycling between the colored states, the color contrast retained almost 100% of its initial value, indicating excellent durability and electrochemical stability. Collectively, the data demonstrate that the violet anthraquinone chromophore combines strong optical modulation and high coloration efficiency, making it a promising material for multicolored electrochromic applications.

## 3. Materials and Methods

### 3.1. General Procedures

All reagents and solvents were obtained from commercial suppliers and used as received. NMR spectra were recorded on a 400 MHz Bruker spectrometer (Madison, WI, USA) with CDCl_3_ as solvent and TMS as reference. High-resolution mass spectrometry data were collected using electrospray ionization (ESI; Synapt G2S Q-TOF, Waters Corporation, Milford, MA, USA). The absorption spectra were measured using quartz cuvettes (1 cm path length).

### 3.2. Synthesis of the Chromophore

The violet anthraquinone derivative was synthesized by condensation between quinizarin and 2-(2-aminoethoxy)ethanol in acetonitrile under an inert atmosphere [40]. (See Supporting Information for full details, including 2D HSQC NMR spectrum).

### 3.3. X-Ray Crystallography

Single-Crystal X-ray data collection was performed at 150 K using a Bruker (Madison, WI, USA) Venture diffractometer equipped with a liquid-gallium Metaljet source, a Helios MX optics and a CMOS Photon III detector. The structure was solved by intrinsic phasing using XT [41] and refined with XL [42]. All the hydrogen atoms linked to carbon atoms were refined using standard riding model, while the hydrogen atoms linked to nitrogen and oxygen were located from difference Fourier map and freely refined with isotropic thermal displacement parameters. CCDC structure deposit 2517348.

### 3.4. Electrochemical Measurements

Electrochemical measurements were done with a potentiostat using a three-electrode cell: glassy carbon working electrode, Pt counter electrode, and Ag^o^ pseudo-reference. Measurements were carried out in anhydrous DMF containing Bu_4_NClO_4_ (0.1 M) at room temperature and the samples were purged with nitrogen for ca. 15 min. before measuring. A gentle blanket of nitrogen was also maintained over the solution during the measurements. Ferrocene was added as an internal reference as the end of the measurements to calibrate the potentials relative to SCE [43].

### 3.5. Spectroelectrochemistry and Colorimetry

Spectroelectrochemical studies were done using a commercial ceramic honeycomb cell (Pine Research Instrumentation, Durham, NC, USA) consisting of 19 gold-plated wells and 0.7 mm thick. The electrode was placed in an optically narrow quartz cuvette (1 mm). Absorption spectra were measured with a continuously applied potential with a potentiostat corresponding to each redox state. The color coordinates (L*, a*, b*) were calculated from the absorption spectra according to CIE 1976 standards.

### 3.6. Theoretical Calculations

Theoretical calculations were done with Gaussian 16 rev. C.01 in a two-step process [44]. First, the geometry was optimized using with the ωB97X-D functional with the def2-TZVP basis set [45]. This was then used as the singlet point energy calculation of TD-DFT to calculate the electronic transitions. The 10 lowest transitions were calculated. For both steps, the IEFPCM solvent model was used with DMF as the solvent. The Natural Transition Orbitals [33] and the lowest-energy electronic transition were also calculated from the TD-DFT [46] data.

## 4. Conclusions

Leveraging the intrinsic double reduction of the anthraquinone concomitant with the oxidation conferred by the electron donor, a molecular electrochrome capable of three discrete color changes was possible. This demonstrates that substituted anthraquinone is ideally suited for electrochromics owing to its reversible electrochemical activity and the subsequent color change with applied potential. Integrating an electron donor, also promoting hydrogen bonding, shifted the absorption of the anthraquinone deep into the visible, enhancing the optical properties for electrochromics. The multiple colors demonstrate the advantage of the molecular anthraquinone compared to its conventional dichromatic electrochromes. Both the color of the neutral and electrochemically mediated intermediates can be tailored by adjusting the anthraquinone substituents. This opens the possibility of tuning their absorptions across the visible toward achieving panchromaticity.

## Figures and Tables

**Table 1:** 1931 CIE color coordinates of **AQ-V** with applied potential.

State	L*	a*	b*
Neutral	67.7	31.9	−47.8
Radical anion	68.7	−20.5	−45.8
Dianion	84.4	15.7	34.5
Cation	100	1.1	4.8

## Data Availability

The data that support the findings of this study are available in the Supporting Information.

## Supplementary Materials

The following supporting information can be downloaded at: https://www.mdpi.com/article/10.3390/molecules31050879/s1, crystallographic, electrochemical, and spectroelectrochemical data, NMR and mass spectrometry spectra, and atomic coordinates from theoretical calculations. Refs. [40,47] are cited in the Supplementary Materials.
