# Supplementary material for: Violet Anthraquinone for Expanding the Color Palette of Electrochromes with Three Discrete Colors and Full Color Bleaching
Source: Molecules. 2026 Mar 6;31(5):879. doi: 10.3390/molecules31050879 (PMC12986092; doi:10.3390/molecules31050879)
Supplement: Supplementary file 1 [file molecules-31-00879-s001.zip › molecules-4081408-supplementary.pdf]

SUPPORTING INFORMATION

Violet Anthraquinone for Expanding the Color Palette of Electrochromes with Three Discrete Colors and full color bleaching

Ilies Seddiki [1], Thierry Maris [2] and W. G. Skene [1,3,*]

[1] Chemistry Department, Université de Montréal, 1375 Avenue Thérèse-Lavoie-Roux, Montreal, QC H2V 0B3, Canada
[2] X-Ray Diffraction Laboratory, Chemistry Department, Université de Montréal, 1375 Avenue Thérèse-Lavoie-Roux, Montreal, QC H2V 0B3, Canada
[3] Institut Courtois, Université de Montréal, Montreal, QC H3C 3J7, Canada
* Correspondence: w.skene@umontreal.ca

Table of Contents

General methods and procedures 3
Synthesis 3
Figure S1. Synthetic scheme for the synthesis of the leuco-quinizarin. 3
Figure S2. Synthetic scheme for the synthesis of **AQ-V**. 4
X-Ray Crystallographic Data 4
Table S1. Hydrogen bond distances and geometries (Å, °) 4
Table S2. Short ring-interactions with Cg-Cg distances[1] 4
Figure S3. Cg1/Cg2/Cg3 (in blue) for 3 aromatics with 6 atoms. 5
Figure S4. Cg4 and Cg5 consisting of cycles with 10 atoms (in red) located on C-C bonds. 5
Figure S5. Cg6 calculated with 14 atoms in proximity to Cg2. 5
Electrochemistry 5
Figure S6. Up to 300 cyclic voltammograms of **AQ-V** measured in anhydrous DMF at 250 mV/sec with Bu_4NClO_4. 6
Figure S7. Cyclic voltammograms **AQ-V** measured at different scan rates in anhydrous DMF with Bu_4NClO_4. 6
Figure S8. Change in cathodic potential of the two reductions and one oxidation of **AQ-V** contingent on scan rate: cation forward wave (black), radical anion forward wave (blue), dianion forward wave dianion (magenta), cation reverse wave (red), radical anion reverse wave (green), and dianion reverse wave dianion (wine). Linear regression for all fits: R^2>0.999 and adjusted R^2>0.999. 7
Spectroelectrochemistry 7
Figure S9. Principal electronic transitions of **AQ-V** calculated by ωB97X-D by TD-DFT. Arrows indicate spectral change between the different states. 8
NMR Spectra 8
Figure S10. ^{1}H-NMR spectrum of **AQ-V** in $CDCl_3$ 8
Figure S11. ^{13}C-NMR spectrum of **AQ-V** in $CDCl_3$ 9
Figure S12. HSQC NMR spectrum of **AQ-V** in $CDCl_3$. 9
Mass Spectrometry 10
Figure S13. HR-MS spectra of **AQ-V**. 10
Atomic Coordinates Theoretical Calculations 10
Table S3. Atomic coordinates of neutral state of **AQ-V** calculated by ωB97X-D with def2-TZVP. 10
Table S4. Atomic coordinates of the radical anion of **AQ-V** calculated by ωB97X-D with def2-TZVP. 11
Table S5. Atomic coordinates of the dianion of **AQ-V** calculated by ωB97X-D with def2-TZVP. 12
Table S6. Atomic coordinates of the radical cation of **AQ-V** calculated by ωB97X-D with def2-TZVP. 13
References .... **Error! Bookmark not defined.**

General methods and procedures

Reagents and solvents were used as received from commercial sources. Solvents for both reactions and characterization measurements were anhydrous and they were obtained from a commercial aluminum column system. Electrochemical measurements were done with a glassy carbon working electrode, platinum wire counter electrode, and silver wire pseudo-reference electrode. Absorption and spectroelectrochemical measurements were done with a combined UV-visible-NIR spectrometer. The working electrode for the spectroelectrochemical measurements was a commercial (Pine Research Instruments) 19 gold well electrode (0.17 mm thick) with a gold counter electrode on a ceramic substrate. A low volume quartz cuvette with an optical pathlength of 1 mm was used.

Synthesis

Leuco-quinizarin [47]. 1,4-Dihydroxyanthraquinone (4 g, 16.7 mmol) and sodium carbonate (4.41 g, 41.6 mmol) were dissolved in water (80 mL). The solution was then heated at 90 °C under nitrogen. Once the mixture was homogenous, sodium hydrosulfite (7.25 g, 41.6 mol) was added and the mixture was stirred for 3 h at 85°C. Afterwards, the solution was cooled to room temperature, filtered, and washed with water (2 x 250 mL). Leuco-quinizarin was obtained as a light brown powder by vacuum drying (3.8 g, 95 %).

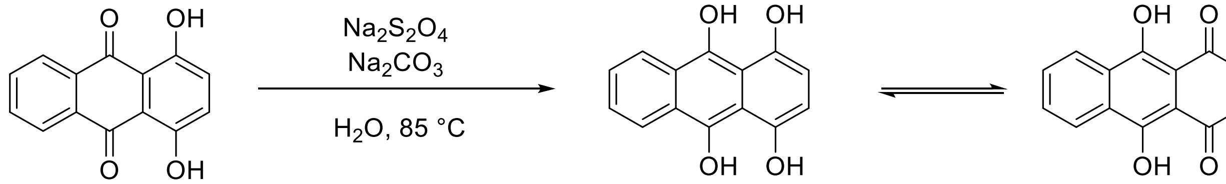

Figure S1. Synthetic scheme for the synthesis of the leuco-quinizarin.

Hydroxy-4-((2-(2-hydroxyethoxy)ethyl)amino)anthracene-9,10-dione (**AQ-V**) [40]. Leuco-quinizarin (3 g, 12.4 mmol) and 2-(2-aminoethoxy)ethanol (3.91 g, 37.2 mmol) were dissolved in anhydrous acetonitrile (70 mL). The solution was heated at 80 °C under nitrogen for 2 days and the reaction was monitored by TLC. After completion, the mixture was cooled to 70 °C under a continuous stream of compressed air. The solvent was removed under vacuum after 1 day. The resulting dark violet viscous oil was taken up in dichloromethane. It was then washed with a saturated solution of water/$NaHCO_3$ (2 x 20 mL) followed by water (2 x 50 mL) to remove the excess of 2-(2-aminoethoxy)ethanol. After removing the solvent by evaporation, the resulting dark violet powder was purified by silica gel column chromatograph (ethyl acetate:hexane (25:75 vol%) to afford **AQ-V** as dark violet powder (1.3 g, 32%). The crude product was purified by column chromatography with ethyl acetate:hexane (25:75 vol%) to yield a dark violet solid (32%). ^{1}H NMR ($CDCl_3$, 400 MHz): δ 13.69 (s, 1H, O-H) 10.57 (s, 1H, N-H), 8.35 (dd, 2H, *J*= 8.5 Hz, Ar-H), 7.78 (dd, 2H, *J*= 7.5 Hz, Ar-H), 7.25 (dd, 2H, *J*= 9,6 Hz, Ar-H), 3.85 (t, 4H; *J*= 3.8 Hz, C-H), 3.74 (t, 2H, *J*= 4.6 Hz, C-H), 3.62 (t, 2H, *J*= 5.2 Hz,
C-H). ^{13}C ($CDCl_3$, 101 MHz): δ 187.5, 182.3, 156.8, 147.7, 135.2, 134.2, 132.7, 132.6, 129.0, 126.9, 126.3, 123.9, 113.8, 108.8, 72.8, 69.4, 62.0, 42.4. HRMS m/z: $[M+H]^+$ Calcd for $C_{18}H_{17}NO_5$ 328.1185; Found 328.1221

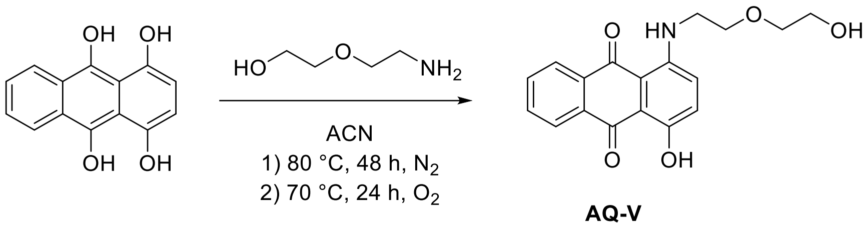

Figure S2. Synthetic scheme for the synthesis of **AQ-V**.

X-Ray Crystallographic Data

Table S1. Hydrogen bond distances and geometries (Å, °)

D—H···*A*	*D*—H	H···*A*	*D*···*A*	*D*—H···*A*
N1—H1···O1	0.902(18)	1.918(17)	2.6207(12)	133.3(14)
O3—H3A···O2	0.956(19)	1.638(19)	2.5284(14)	153.2(17)
O5—H5···O3^{i}	0.903(19)	2.27(2)	3.1126(14)	154.7(16)
C15—H15A···O2^{ii}	0.99	2.66	3.1432(14)	110.5
C18—H18B···O1^{iii}	0.99	2.60	3.3128(15)	129.0

Symmetry codes: (i) *x*+1/2, −*y*+1/2, *z*+1/2; (ii) *x*+3/2, −*y*+1/2, *z*+1/2; (iii) −*x*+2, −*y*+1, −*z*+1.

Table S2. Short ring-interactions with Cg-Cg distances[1]

**Cg (I)**	**Cg (J)**	**Symm J**	**Cg···Cg (Å)**	**α (°)**	**β (°)**	**γ (°)**	**CgI Perp. (Å)**	**CgJ Perp. (Å)**	**Slippage (Å)**
Cg1	Cg2	1655	3.8379	1.30	28.9	27.7	3.3970	3.3604	1.854
Cg2	Cg1	1455	3.8379	1.30	27.7	28.9	3.3604	3.3971	1.786
Cg3	Cg2	1455	3.8755	1.69	26.2	27.1	3.4506	3.4780	1.710
Cg2	Cg3	1655	3.8755	1.69	27.1	26.2	3.4779	3.4506	1.764
Cg1	Cg1	1455	4.4600	0.03	41.2	41.2	3.3578	3.3577	2.936
Cg1	Cg1	1655	4.4600	0.03	41.2	41.2	3.3577	3.3577	2.936
Cg2	Cg2	1455	4.4600	0.00	40.1	40.1	3.4139	3.4140	2.870
Cg2	Cg2	1655	4.4600	0.00	40.1	40.1	3.4140	3.4139	2.870
Cg3	Cg3	1455	4.4600	0.00	38.4	38.4	3.4969	3.4969	2.768
Cg3	Cg3	1655	4.4600	0.00	38.4	38.4	3.4969	3.4969	2.768
Cg1	Cg3	1655	4.7021	2.89	43.4	43.6	3.4057	3.4170	3.230
Cg3	Cg1	1455	4.7021	2.89	43.6	43.4	3.4170	3.4057	3.242

[1] Distances < 5.0 Å, α < 20.000 ° and β < 60.0 °.
Cg refer to the Center of gravity of rings:
Cg1: C1 C2 C3 C4 C5 C14
Cg2: C5 C6 C7 C12 C13 C14
Cg3: C7 C8 C9 C10 C11 C12

π–π stacking between ring centroids (Cg) with symmetry 1455 = −1+X, Y, Z; 1655 = 1+X, Y, Z. Closest stacks are Cg1 with Cg2 (1655) and Cg3 with Cg2 (1655), with Cg···Cg = 3.8379(9)

& 3.8755(9) Å, α = 1.30(5) & 1.69(5)°, β ≈ 26 & 29°, perpendicular separations = 3.39 & 3.42 Å, and slippage ≈ 1.71 & 1.85 Å.

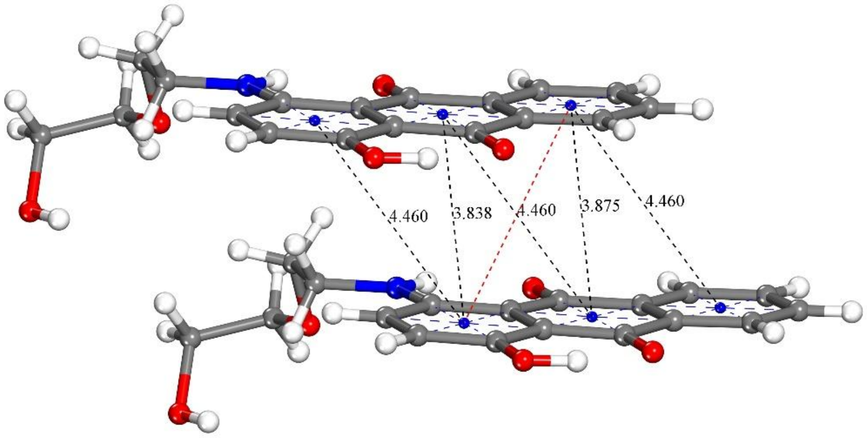

Figure S3. Cg1/Cg2/Cg3 (in blue) for 3 aromatics with 6 atoms.

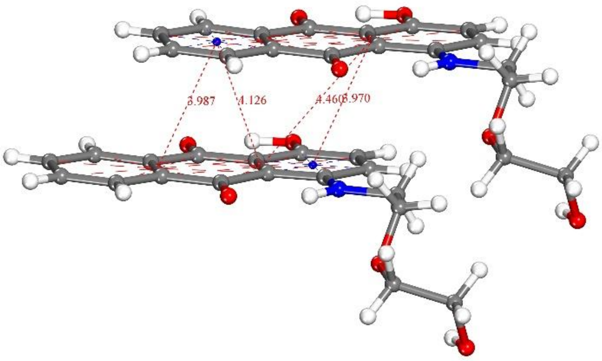

Figure S4. Cg4 and Cg5 consisting of cycles with 10 atoms (in red) located on C-C bonds.

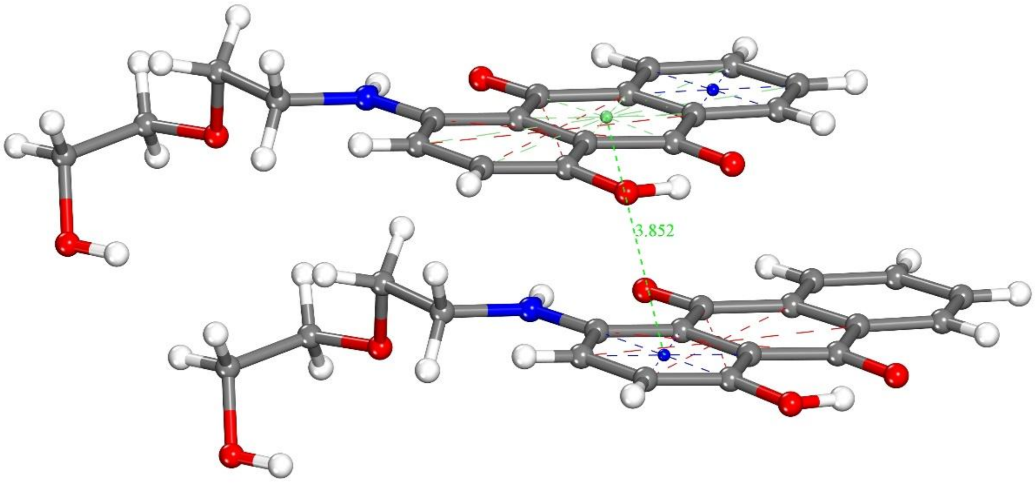

Figure S5. Cg6 calculated with 14 atoms in proximity to Cg2.

Electrochemistry

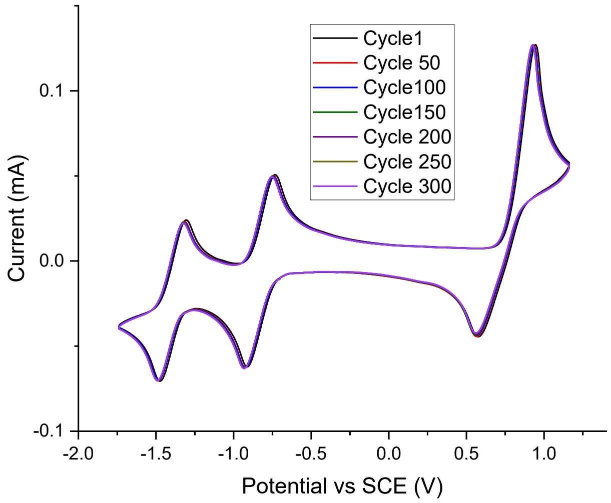

Figure S6. Up to 300 cyclic voltammograms of **AQ-V** measured in anhydrous DMF at 250 mV/sec with Bu_4NClO_4.

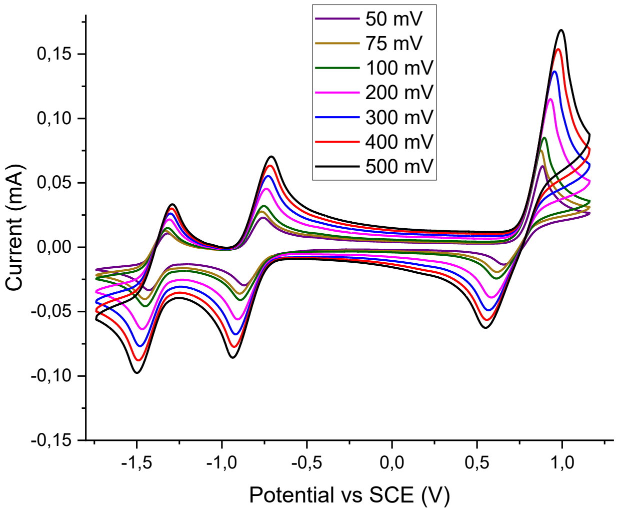

Figure S7. Cyclic voltammograms **AQ-V** measured at different scan rates in anhydrous DMF with Bu_4NClO_4.

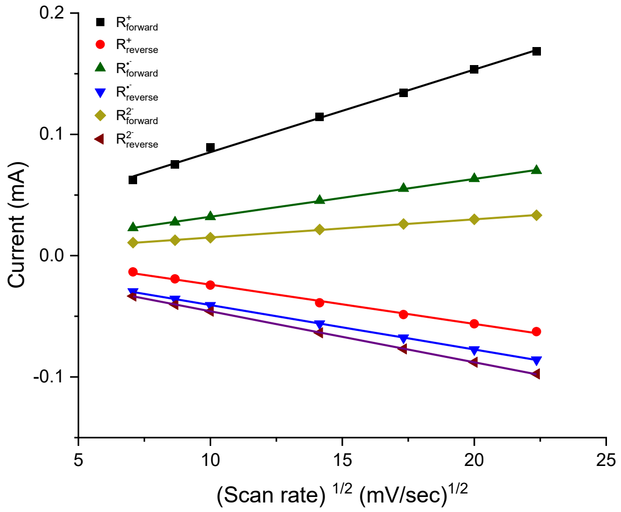

Figure S8. Change in cathodic potential of the two reductions and one oxidation of **AQ-V** contingent on scan rate: cation forward wave (black), radical anion forward wave (blue), dianion forward wave dianion (magenta), cation reverse wave (red), radical anion reverse wave (green), and dianion reverse wave dianion (wine). Linear regression for all fits: R^2>0.999 and adjusted R^2>0.999.

Spectroelectrochemistry

The coloration efficiency was calculated according to:

$$CE = \frac{\Delta OD}{Q} = \frac{\log\left(\frac{T_b}{T_c}\right)}{Q} \; Equation\ S1$$

where ΔOD is the change in optical density, T_b and T_c are the transmissions as the bleached and colored states, respectively, and Q is the charge density (C cm^{-2}).

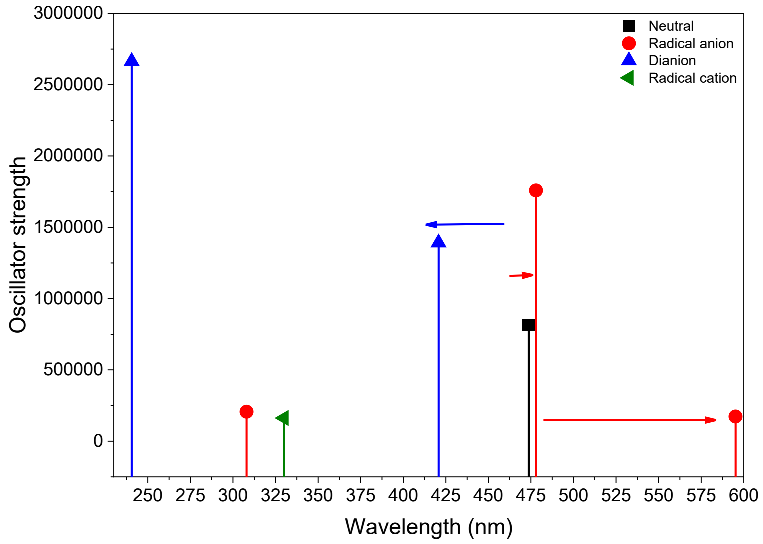

Figure S9. Principal electronic transitions of **AQ-V** calculated by ωB97X-D by TD-DFT. Arrows indicate spectral change between the different states.

NMR Spectra

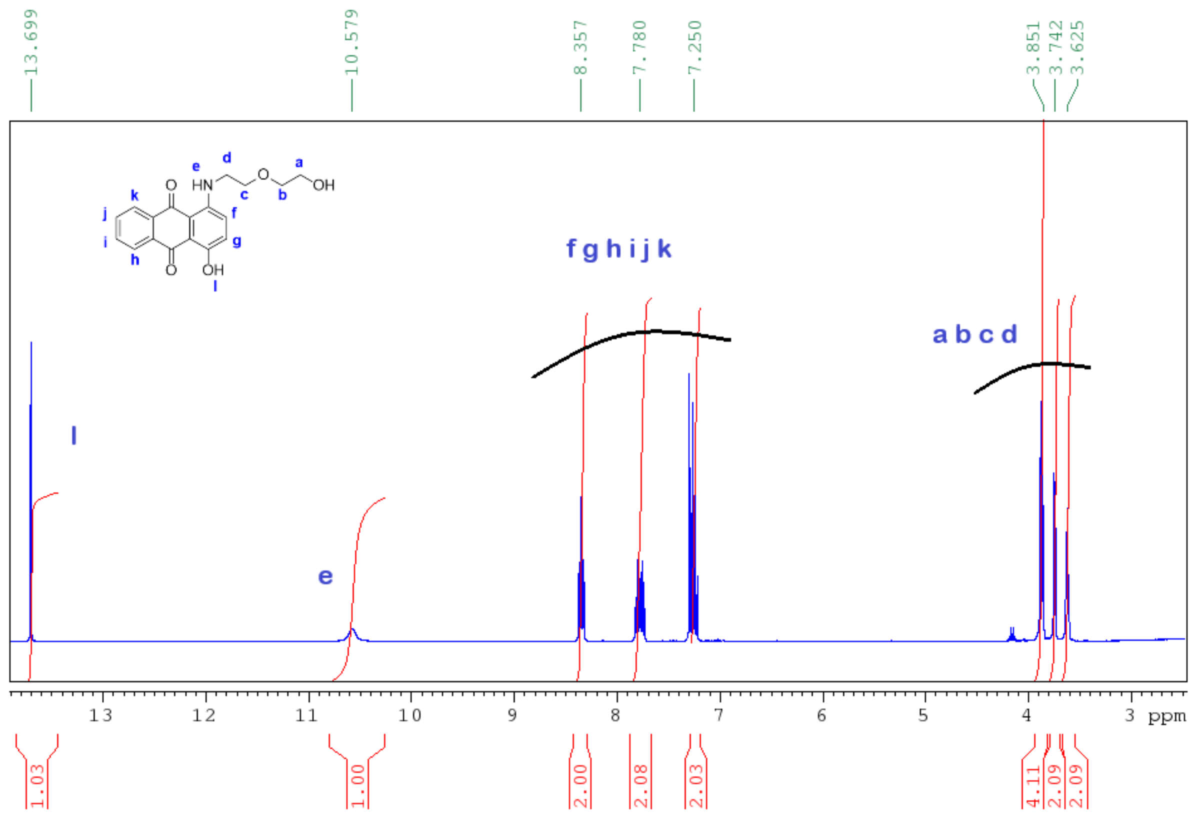

Figure S10. ^{1}H-NMR spectrum of **AQ-V** in $CDCl_3$.

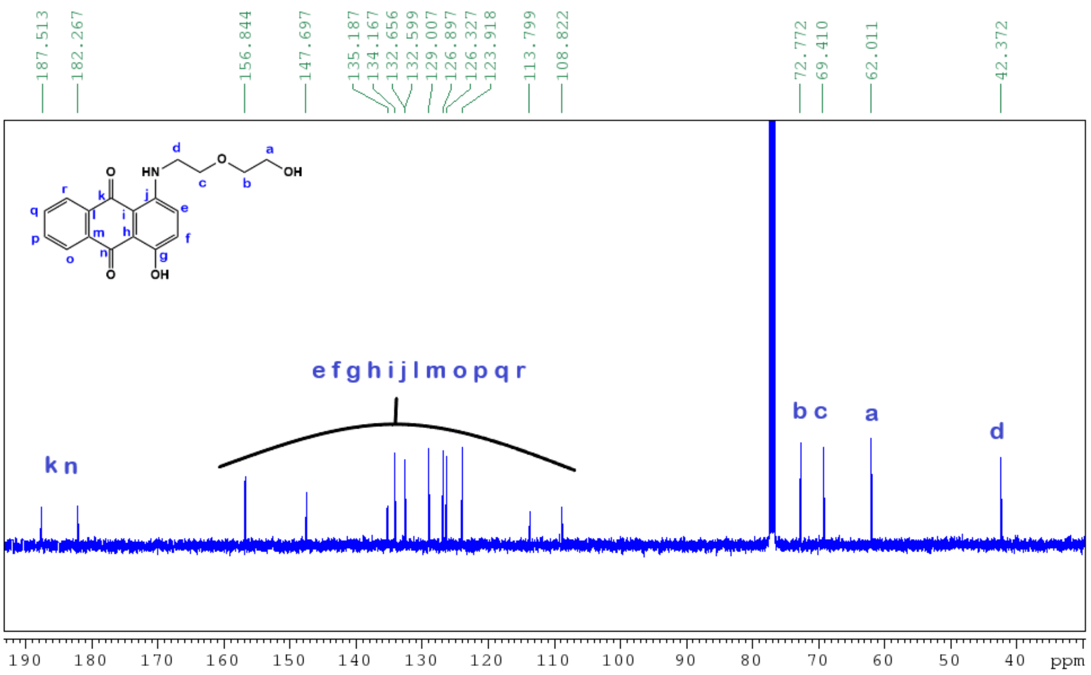

Figure S11. ^{13}C-NMR spectrum of **AQ-V** in $CDCl_3$.

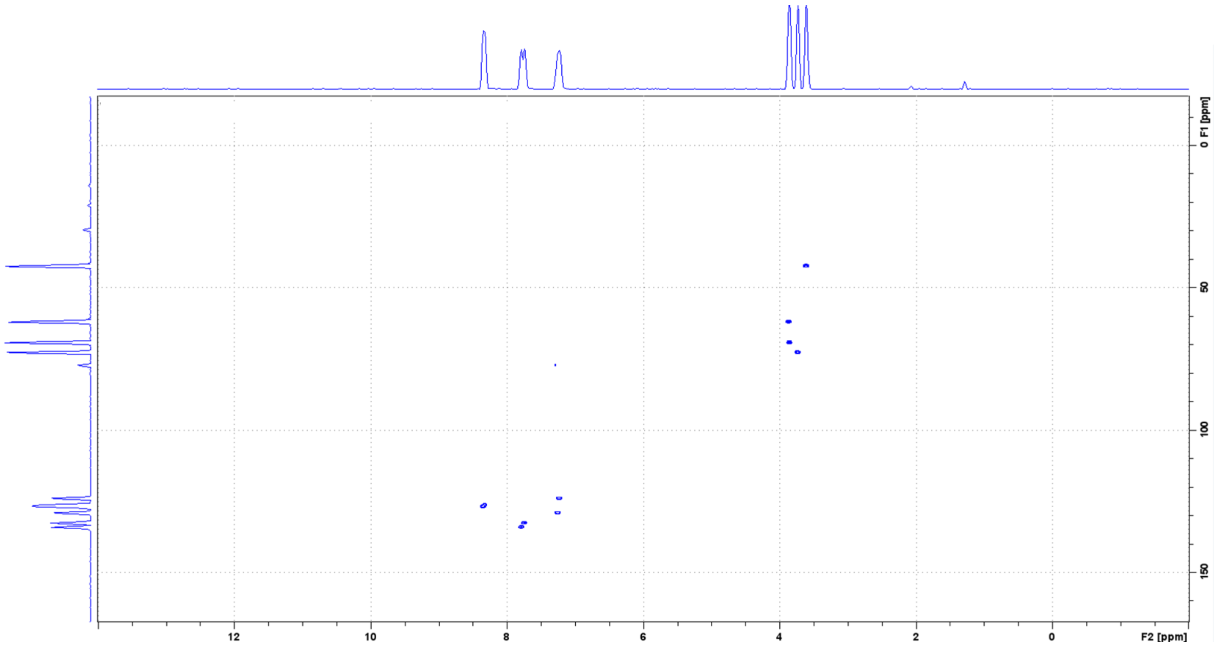

Figure S12. HSQC NMR spectrum of **AQ-V** in $CDCl_3$.

Mass Spectrometry

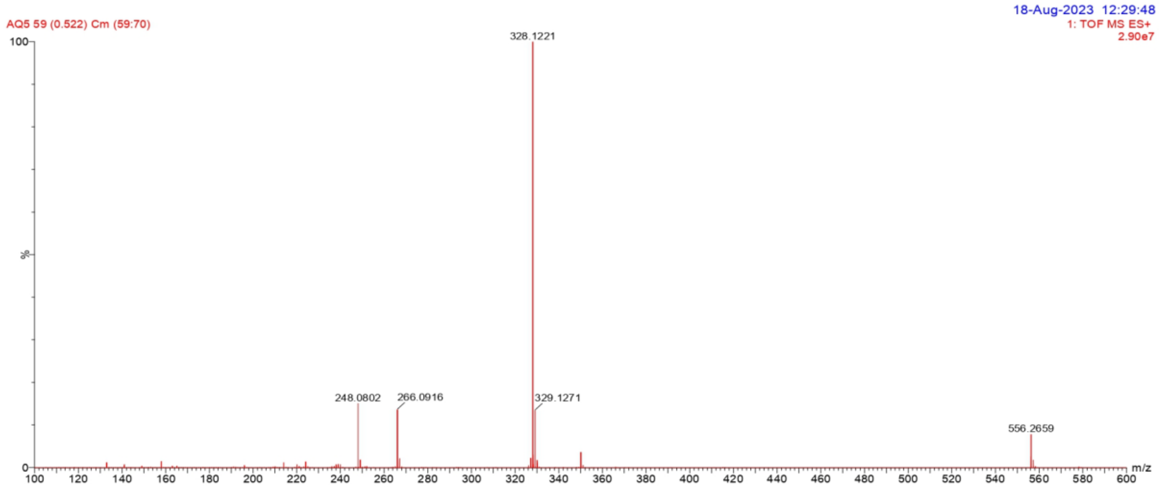

MS spectrum (Zoom)

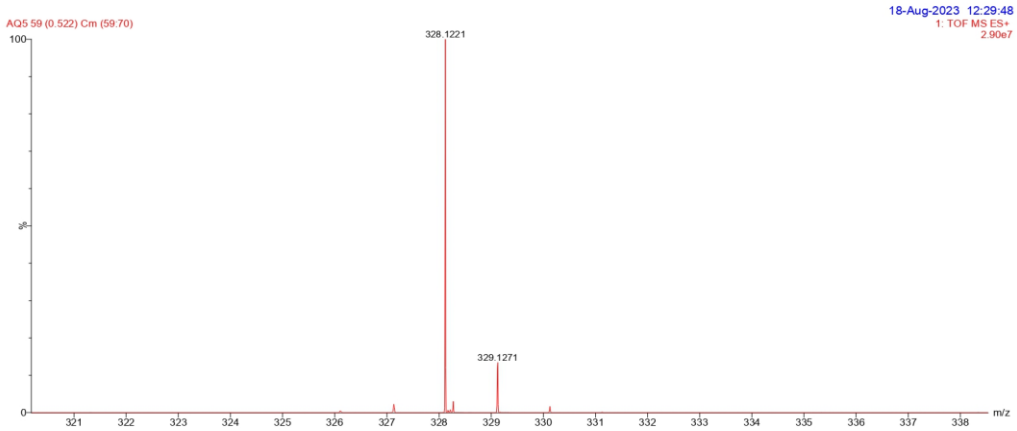

Ion	formula	Expe. m/z	Calc. m/z	Diff (mDa)
[M+H]+	C18H17NO5	328.1221	328.1185	-3.6

Figure S13. HR-MS spectra of **AQ-V**.

Atomic Coordinates Theoretical Calculations

Table S3. Atomic coordinates of neutral state of **AQ-V** calculated by ωB97X-D with def2-TZVP.

Atom	X	Y	x
C	-4.30655	-0.26631	-0.00061
C	-3.34649	0.72746	-0.0003
C	-1.99526	0.39223	0.00017
C	-1.60682	-0.94693	0.00029
C	-2.58047	-1.94057	-0.00003
C	-3.92145	-1.60287	-0.00047

C	-0.9785	1.46124	0.00053
C	-0.16965	-1.33899	0.00073
C	0.83506	-0.28973	0.00031
C	0.43209	1.08445	0.00031
C	1.38588	2.09661	0.00006
C	2.75284	1.75603	-0.00024
C	3.14907	0.4576	-0.00028
C	2.21014	-0.61391	-0.00004
H	-5.35649	-0.00429	-0.00097
H	-3.62779	1.7716	-0.0004
H	-2.26961	-2.97606	0.00007
H	-4.67291	-2.38185	-0.00072
H	3.48175	2.55609	-0.00037
H	4.20598	0.233	-0.00047
O	0.11331	-2.53922	0.00008
O	-1.33198	2.64531	-0.00052
N	2.65659	-1.88383	-0.00022
H	1.93847	-2.59288	0.0006
O	1.08404	3.39768	0.00023
H	0.09886	3.45117	0.00076
C	4.04706	-2.26691	0.0002
H	4.57109	-1.90197	0.88771
H	4.09822	-3.3528	-0.00002
H	4.57165	-1.90165	-0.88682

Table S4. Atomic coordinates of the radical anion of **AQ-V** calculated by ωB97X-D with def2-TZVP.

Atom	X	Y	x
C	-4.28188	-0.28484	0.0003
C	-3.33504	0.70604	0.00013
C	-1.96199	0.39353	-0.00005
C	-1.56463	-0.95661	-0.00005
C	-2.55294	-1.95672	0.00017
C	-3.88622	-1.63452	0.00032
C	-0.98316	1.45779	-0.00009
C	-0.15825	-1.34711	-0.00025
C	0.82613	-0.28212	-0.00096
C	0.41194	1.08544	-0.0004
C	1.3873	2.11998	0.00008
C	2.72461	1.7938	0.00025
C	3.14078	0.46327	-0.00049
C	2.22594	-0.58349	-0.00152
H	-5.33585	-0.03	0.00043
H	-3.61051	1.75276	0.00015
H	-2.21957	-2.98645	0.00019

H	-4.63549	-2.41823	0.00045
H	3.45103	2.59768	0.0009
H	4.20141	0.24871	-0.00061
O	0.13864	-2.57026	0.00036
O	-1.35656	2.67159	0.00019
N	2.64466	-1.8868	-0.00362
H	1.88829	-2.56399	-0.00016
O	1.02794	3.41749	0.00058
H	0.02642	3.39969	0.00054
C	4.01567	-2.2705	0.00275
H	4.55411	-1.90362	0.88945
H	4.07607	-3.35909	0.00308
H	4.56171	-1.90405	-0.87935

Table S5. Atomic coordinates of the dianion of **AQ-V** calculated by ωB97X-D with def2-TZVP.

Atom	X	Y	x
C	-4.2642	-0.3245	-0.0013
C	-3.3354	0.67219	-0.012
C	-1.9369	0.38526	-0.0121
C	-1.521	-0.9813	0.00168
C	-2.5218	-1.985	0.01594
C	-3.8538	-1.6827	0.01183
C	-0.9791	1.40103	-0.0106
C	-0.1338	-1.3557	0.01179
C	0.82598	-0.2867	-0.0472
C	0.38909	1.0857	-0.0227
C	1.36016	2.1847	0.01355
C	2.69294	1.84657	0.03732
C	3.13258	0.49661	-0.0178
C	2.25301	-0.5511	-0.0917
H	-5.3208	-0.0819	-0.0029
H	-3.6457	1.70921	-0.0209
H	-2.1897	-3.0154	0.02865
H	-4.5966	-2.4716	0.01842
H	3.4258	2.64635	0.08456
H	4.19843	0.30342	-0.0371
O	0.1885	-2.6035	0.08249
O	-1.3946	2.70096	0.01075
N	2.69275	-1.8667	-0.2602
H	1.9434	-2.515	-0.0259
O	0.93543	3.43086	0.03428

H	-0.4983	3.21469	0.02138
C	4.02681	-2.2278	0.1304
H	4.29318	-1.8778	1.14086
H	4.12114	-3.3139	0.10799
H	4.77707	-1.8203	-0.5561

Table S6. Atomic coordinates of the radical cation of **AQ-V** calculated by ωB97X-D with def2-TZVP.

Atom	X	Y	x
C	-4.3202	-0.2159	-0.0004
C	-3.3472	0.76832	-0.0003
C	-2.0046	0.41138	-8E-05
C	-1.637	-0.9352	0
C	-2.6194	-1.9171	-0.0001
C	-3.9561	-1.5562	-0.0003
C	-0.9751	1.4589	0
C	-0.2158	-1.3422	0.00026
C	0.82744	-0.2767	0.00014
C	0.45121	1.05614	0.00006
C	1.44036	2.07689	0.00002
C	2.81628	1.73607	0.00011
C	3.197	0.443	0.00016
C	2.22852	-0.6119	0.00015
H	-5.3658	0.06099	-0.0005
H	-3.617	1.81521	-0.0003
H	-2.3273	-2.9578	-4E-05
H	-4.7188	-2.3234	-0.0004
H	3.54078	2.5384	0.00011
H	4.24744	0.19322	0.00025
O	0.08755	-2.5228	0.00005
O	-1.2778	2.64636	-3E-05
N	2.62251	-1.8705	0.00018
H	1.86516	-2.5545	0.00034
O	1.13755	3.34367	-0.0001
H	0.14436	3.41477	-0.0003
C	3.99458	-2.3356	0.00016
H	4.51717	-1.9854	0.89037
H	3.98005	-3.4203	-0.0004
H	4.51747	-1.9845	-0.8895
